# Human hair as a diagnostic tool in medicine

**DOI:** 10.1016/j.bbrep.2025.102129

**Published:** 2025-07-05

**Authors:** Venetia A. Florou, Auraya Manaprasertsak, Maria Slyusarenko, Sarah R. Amend, Julhash U. Kazi, Emma U. Hammarlund, Kenneth J. Pienta

**Affiliations:** a The Cancer Ecology Center, The Brady Urological Institute, Johns Hopkins School of Medicine, Baltimore, MD, 21287, USA; b Department of Experimental Medical Science, Lund University, Lund, Sweden; c Lund University Cancer Center (LUCC) and Lund Stem Cell Center (SCC), Lund University, Lund, Sweden; d Division of Translational Cancer Research, Department of Laboratory Medicine, Lund University, Lund, Sweden

**Keywords:** Hair analysis, Heavy metals, Biomarkers

## Abstract

Hair analysis has been used in the past as a method to explore environmental exposure to chemicals, toxins or drug ingestion, with wide applications in forensic science, environmental studies, anthropology and medicine. Although not a conventional sample type, hair offers significant advantages over traditional biospecimens, such as blood or urine. These include its stability, non-invasive nature and ease of collection and handling, as well as its ability to provide a “fossilized” record of a person's health or living conditions. These unique characteristics, along with advancements in analytical techniques have enabled hair analysis as a promising tool for the development of new biomarkers for both prognostic and diagnostic purposes in a range of medical conditions. The new developments in hair analysis add to its recognized diagnostic value in fields such as toxicology and pharmacology. In this review, we summarize recent data, ongoing developments and emerging possibilities of hair analysis as a scientific and diagnostic tool.

## Highlights

• Hair has the potential to serve as a valuable biomarker due to its easy, non-invasive collection.
• A chronological record of body changes can be captured through hair over extended periods.
• Distinct heavy metal accumulation patterns have been found to correlate significantly with various cancer types.
• Hair analysis potentially offers an easily accessible tool for population screening for many diseases.

## 1 Introduction

The use of biological samples to diagnose, detect, and monitor acute and chronic diseases has revolutionized medical practice. Samples of blood, urine, stool, saliva and tissue are used routinely in clinical practice as vehicles to measure analytes or to study biomarkers. These biospecimens, particularly blood and urine, provide real-time snapshots of ongoing or recent biological activity. Their application, however, is limited by the requirements of sterile collection and the need for preservatives, which limit the time frame in which they can be utilized. In liquid samples, the molecule of interest is also generally more dilute than in tissues and keratin-rich structures, such as hair and nails.

Hair as a medium for biomarker analysis has been used for years by environmental and forensic sciences, as well as anthropology and medicine to study exposure to environmental compounds, toxins, or drugs (Table 1). As hair grows continuously at approximately 1 cm a month, it essentially provides a chronological “archive” of physiological changes and exposures over extended periods [1,2]. These unique characteristics underscore the significance of hair as an investigative and diagnostic tool to monitor health. Furthermore, due to the recording system, hair can reveal evidence of chronic exposures or disease processes that can be missed by one-time blood tests. Moreover, hair collection is non-invasive, requires no special handling or refrigeration, and hair samples are stable over long periods, making them convenient for longitudinal monitoring and even retrospective analysis of stored samples.

**Table 1:**
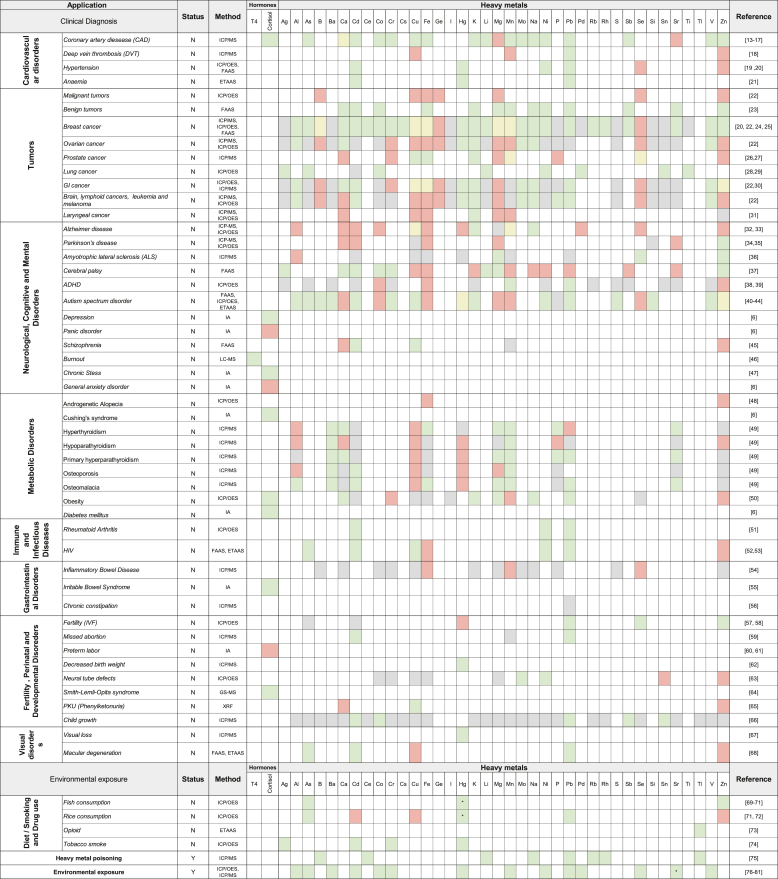
Hair analysis as a tool in medical diagnostics. *A positive correlation between an element and a disorder is shown in green, while a negative correlation is shown in red. Evidence about both positive and negative correlation between an element and a disorder are marked in yellow. Elements that were tested but showed no difference between the group of interest and the control group are marked in gray; An asterisk (∗)* indicates that specific isotopes of the respective element were tested; “Status” describes whether the tested substance or drug is routinely used in clinical practice (Y: Yes; N: No)*; **ICP/MS:**Inductively Coupled Plasma Mass Spectrometry****; ICP/OES:****Inductively Coupled Plasma Optical Emission Spectroscopy****; FAAS:****Flame Atomic Absorption Spectrophotometry****; ETAAS:****Electrothermal Atomic Absorption Spectrometry****; GC-MS:****Gas Chromatography-Mass Spectrometry****; IA:****Immunoassay****; LC-MS:****Liquid Chromatography-Mass Spectrometry****; T4:****Thyroxine****; Ag****– Silver;****Al****– Aluminum;****As****– Arsenic;****B****– Boron;****Ba****– Barium;****Ca****– Calcium;****Cd****– Cadmium;****Ce****– Cerium;****Co****– Cobalt;****Cr****– Chromium;****Cs****– Cesium;****Cu****– Copper;****Fe****– Iron;****Ge****– Germanium;****I****– Iodine;****Hg****– Mercury;****K****– Potassium;****Li****– Lithium;****Mg****– Magnesium;****Mn****– Manganese;****Mo****– Molybdenum;****Na****– Sodium;****Ni****– Nickel;****P****– Phosphorus;****Pb****– Lead;****Pd****– Palladium;****Rb****– Rubidium;****Rh****– Rhodium;****S****– Sulphur;****Sb****– Antimony;****Se****– Selenium;****Si****– Silicon;****Sn****– Tin;****Sr****– Strontium;****Ti****– Titanium;****Tl****– Thallium;****V****– Vanadium;****Zn****– Zinc* [[17], [19], [20], [21], [27], [31], [32], [33], [34], [35], [36], [37], [38], [39], [40], [41], [42], [43], [44], [45], [46], [48], [49], [50], [51], [52], [53], [56], [57], [58], [59], [60], [61], [62], [63], [64], [65], [66], [67], [68], [69], [70], [72], [73], [74], [75], [76], [77], [78], [79]].

Traditionally, hair has been extensively used in forensic science and environmental toxicology to assess exposure to drugs, poisons, and pollutants. As early as the mid-19th century, arsenic was identified in hair from an exhumed body 11 years after death [3] highlighting the value of hair in detecting long-past exposures. In forensic casework, hair analysis became a robust tool throughout the 20^th^ century for documenting drug use or toxin exposure when other evidence had degraded [4,5].

Despite this success in forensics, hair has seen limited use in routine medical diagnostics. One reason is that biochemical readout of hair samples is an averaged signal over time, which can reduce sensitivity to rapid changes. Clinicians have traditionally relied on blood or urine for their immediacy in reflecting current physiological states. However, there is growing recognition of the clinical value of the ability of hair samples to reflect chronic exposures and long-term physiological states. By providing an integrated measure of biochemical status, hair can complement acute markers and potentially enable earlier detection of slowly developing conditions. For instance, accumulating evidence links hair measurements, such as cortisol or trace elements, with chronic stress and disease risk [6,7], suggesting hair analysis could serve as an early warning or monitoring tool for conditions that develop over months or years. In addition, the ease and non-invasiveness of hair sampling make it appealing for population screening and for patients where frequent blood draws are impractical. Yet, there are limitations to and of the use of hair in clinical practice. This review summarizes current applications of hair as a scientific and diagnostic tool as well as potential future directions that may integrate hair into clinical practice.

### 1.1 Hair anatomy and biology

**Fig. 1:**
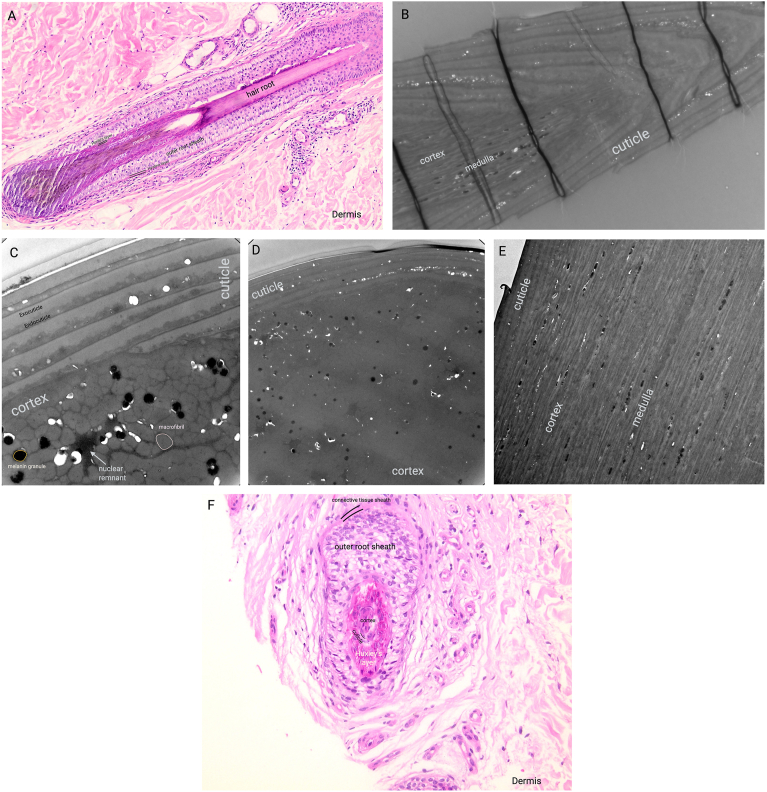
Hair anatomy; (A) Longitudinal section of hair follicle and hair root: H&E staining; (B) Longitudinal section of hair strand using Transmission Electron Microscopy (TEM); (C) High magnification of cross-section of hair strand (D)- TEM imaging; (E): High magnification of longitudinal section of hair strand illustrating the cuticle, cortex, and medulla in detail- TEM imaging; (F): H&E staining of hair shaft in cross-section.

Understanding the structure and biology of hair is essential for appreciating how biomarkers are incorporated and what information can be gleaned from hair analyses. Hair evolved to play a critical role in thermoregulation, while also protecting the skin against ultraviolet radiation and trauma [8,9]. Hair's function is supported by a structure composed of two main components: the root and the shaft. The hair root stems from the hair follicle and the hair shaft extends above the skin surface (Fig. 1). The follicle produces the cortical cells, which adhere together as they grow and form the main body of the hair shaft. In turn, the hair shaft can be divided into three layers, best observed in a cross-sectional view: the cuticle, the cortex, and the medulla (Fig. 1).

The cuticle forms the hair's outer layer, surrounding and protecting the cortex. The cuticle is comprised of layers of flat overlapping cells, oriented from the proximal to the distal end of the hair fiber. The largest compartment of the hair fiber is the cortex. The cortex consists of long cortical cells that are constructed from intermediate filaments. The filaments are organized into macrofibrils that are connected to each other via the cell membrane complex. The cortex contains the majority of hair's structural proteins, providing strength and elasticity. The structural building blocks of the cortex include twelve types of keratins, accounting for one-third of the cortex's protein content, and more than 100 keratin-associated proteins (KAPs), making up the remaining protein. KAPs are classified into three large categories based on their amino-acid composition: high-sulphur, ultra-high-sulphur, and high glycine or threonine proteins [[10], [11], [12],^82^]. The medulla, when present, is found at the core of the hair shaft. The medulla is composed of the cell membrane complex, intermixed with medullary and cortical cells and also contains lipids, water, and small amounts of minerals [4].

Hair growth occurs in a cyclical process with three main phases, anagen, catagen, and telogen. In the anagen phase, cells in the hair follicle divide, adding to the hair shaft at the rate of approximately 1 cm a month. During this growth phase, the follicle incorporates nutrients, elements, and other necessary factors to support growth. Simultaneously, it also incorporates other substances from the blood. This forms the basis for many applications of hair analysis. The catagen phase is a transition phase that lasts approximately two weeks. In this phase, hair growth slows down and the follicle shrinks. In the telogen phase, generally lasting several months, the hair shaft is shed, and a new hair begins to grow. Approximately 10 % of scalp hair is in the telogen phase at any given time [2,83,84].

### 1.2 Hair analysis as a tool in medical diagnostics

#### 1.2.1 Drug and alcohol testing

One of the most well-established applications of hair analysis is in the detection and monitoring drug use, both in forensic investigations and clinical settings. Unlike blood or urine, hair provides a long-term retrospective record of substance exposure, with detection windows extending from weeks to several months following ingestion. Given that scalp hair grows at an average rate of approximately 1 cm per month, segmental hair analysis allows for temporal resolution of drug intake patterns, offering insights into the timing, frequency, and duration of drug use or abstinence. For instance, a person who stopped using a drug two months ago will show lower concentrations (or none) in the 1-cm segment closest to the scalp compared to more distal segments that correspond to earlier use [2,5,85]. This temporal resolution makes hair testing invaluable for distinguishing new use from past use and for verifying claims of abstinence or relapse in drug rehabilitation programs.

The rate and extent of drug incorporation into hair are multifactorial and depend both on the chemical properties of the substance (such as lipophilicity) and the physical characteristics of the hair strand (such as melanin content) [169]. Drug uptake is facilitated by the hair root, as substances passively diffuse from the blood through the keratin matrix. Other routes of drug absorption include sweat, as well as apocrine and sebaceous glands. As the environment around and inside the follicle contains lipid-rich components (such as sebum, lipid membranes etc), it is easier for lipophilic drugs to diffuse into the developing hair. Once incorporated, lipophilic drugs, such as cocaine have higher affinity for hydrophobic regions of keratins compared to hydrophilic drugs. In addition, the pH difference between the blood and the hair root environment facilitates the diffusion of basic drugs, resulting in higher accumulation compared to acidic drugs. Notably, the concentration of incorporated drugs is also influenced by the amount of melanin found in the strand. Since melanin has acidic functional groups, basic drugs, such as cocaine and amphetamines, form inonic bonds with melanin and tend to bind stronger than acidic drugs [170]. Therefore, drugs that are both lipophilic and basic can more readily incorporate inside the hair strand and remain longer in pigmented compared to less pigmented hair [86,87,105,106,169,170].

Testing hair to detect drug use was first popularized in the late 1970s. In 1979, investigators successfully detected heroin use through hair analysis, initially using radioimmunoassay techniques [88]. Over time, more specific techniques like GC-MS and LC-MS became the gold standard, allowing not only the detection of parent drugs but also unique metabolites that confirm the source of the drug. For example, the presence of 6-acetylmorphine in hair is a marker of heroin (diacetylmorphine) use, distinguishing it from morphine taken by other routes [89]. In addition, other heroin byproducts such as acetylcodeine, benzoylecgonine or cocaethylene can aid in differentiating between medically prescribed and illicit heroin [5]. Since then, other opioids such as morphine, codeine, oxycodeine and their metabolites can be routinely examined for clinical or forensic purposes. Additionally, hair can be routinely tested for exposure to amphetamines or the street drug phencyclidine [[90], [91], [92], [93]]. Forensic applications of hair drug testing include workplace drug testing programs, criminal justice cases such as, verifying drug use history of a suspect, and post-mortem toxicology as hair can reveal drug use history even when blood and organs no longer contain the drugs due to metabolism or decomposition.

Clinically, hair drug testing is used for monitoring compliance or substance abuse in certain scenarios. A notable application is in identifying chronic alcohol and cannabis use. While ethanol itself is not incorporated into hair in significant amounts, ethanol metabolites like ethyl glucuronide (EtG) and fatty acid ethyl esters (FAEEs) are deposited in hair and serve as robust markers of alcohol consumption [94]. The EtG and FAEEs hair concentrations are more sensitive and specific for monitoring drinking behavior compared to other alcohol biomarkers and can accurately distinguish between excessive drinkers and abstainers or social drinkers [5,95]. In fact, segmental hair analysis can even differentiate patterns of drinking, for example, consistent daily drinking vs. episodic binge drinking, by the distribution of alcohol metabolites along the hair length [95]. Clinically, this is useful in contexts such as liver transplant candidacy evaluations, monitoring patients in alcohol treatment programs, or investigating fetal alcohol exposure via analyzing maternal hair segments corresponding to each trimester of pregnancy, as well as neonatal hair. Chronic alcohol abuse in this scenario can result in severe implications to the embryo causing a wide range of cognitive and physical abnormalities [96]. Cannabis use can be indicated by the presence of tetrahydrocannabinol (THC) metabolites in hair [97], although cannabinoid detection in hair is less sensitive and still an area of active research due to issues with environmental smoke contamination and lower incorporation rates.

**Fig. 2:**
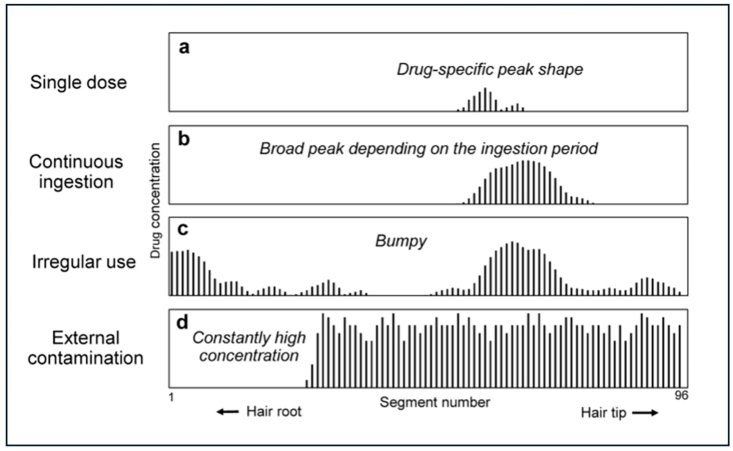
Different distribution patterns obtained by micro-segmental hair analysis, depending on the frequency and type of drug use. The curves were generated after dissecting the hair samples into 0.4 mm segments and analyzing them independently. Each segment represents one day of hair growth. The two peaks observed in each curve represent the two routes of drug absorption, namely via the bloodstream or via sweat and sebaceous glands. Adapted from Kuwayama et al. [101].

Hair analysis also plays a role in forensic medicine for cases of drug-facilitated crimes as well as the potential for compliance monitoring in medical settings. In drug-facilitated assault cases, the victim's hair can be analyzed weeks after an incident to detect drugs like sedatives or hypnotics that have short detection windows in urine or blood. For example, a single high dose of a benzodiazepine or sedative may vanish from blood in hours, but a hair sample taken a few weeks later and segmented to the time of the incident can confirm exposure. These high-resolution, time-specific results can be achieved through micro-segmental hair analysis (MSA), in which the hair strand is dissected into 0.4 mm segments each corresponding approximately to one day of hair growth, and each segment is analyzed independently. This technique allows temporal estimation of a drug ingestion by measuring the distance between the hair root and the proximal drug peak (Fig. 2) [98,101]. Since hair growth rates can vary between individuals, different body parts or even between different hair strands collected from the same area, this estimation may not reflect the exact day of intake in all cases. For this reason, the use of internal temporal markers is necessary in order to determine the timescale of the injected drugs within individual hair strands. [101]. MSA also offers the ability to differentiate the route of drug intake, as hematogenous absorption is indicated by a proximal peak while uptake from sebus or sweat is represented by a distal peak (Fig. 2). Different drugs may exhibit a dominant proximal or distal peak based on their excretion profile. For example, diphenhydramine could be successfully detected in hair strands and the day of ingestion could be identified using this method [99,100].

There have been cases where infant or child poisoning was uncovered by hair tests: one case detected the antipsychotic drug Zuclopenthixol in hair collected from a 2 year old child, identifying a single episode of drug administration that would have been hard to prove otherwise [101]. In pain management or psychiatric treatment, hair testing has been explored to verify if patients are taking their medications or diverting them; for example, hair can be tested for chronic use of opioids or antidepressants as an indication of compliance over time, although routine use of this in practice is still limited [102,103].

Despite the evident advantage of hair to incorporate drugs over long periods, the complexity of its structure requires a multi-step sample preparation to achieve accurate results. Following collection, the hair sample undergoes one or more washing steps to decontaminate the outer part of the hair from external contamination. Different approaches have been developed to ensure effective decontamination from the outer hair layer, while preserving substances incorporated in the inner layers. The most commonly used washing steps, which can be applied to all drug classes, include the use of either dichloromethane or methanol, while isopropanol is primarily used for cannabidoids. Following the appropriate washing steps, the sample is segmented in 1-3 mm pieces and pulverized using a ball mill to increase the surface area for the maximum solvent penetration [104].

The next step in hair analysis includes the separation of the drug of interest from the hair keratinous matrix using the appropriate extraction method. The extraction efficiency is a critical step for reliably quantifying the drug of interest. Thus, the chemical properties of the analyte and the possible interactions between the solvent used during the extraction process should be carefully evaluated. There are three main categories of analyte extraction methods: chemical digestion methods, solvent extraction methods and mechanical-assisted methods. Chemical digestion methods rely on breaking down the keratin matrix to release the incorporated analytes and can be performed by enzymatic, acidic or basic approaches. In enzymatic digestion, enzymes such as pronase and proteinase K are used under neutral pH conditions to break down the hair structure without affecting the extracting drug. This method is especially useful for drugs that are sensitive to strong pH conditions. On the other hand, acidic extraction involves strong acids such as hydrochloric acid (HCl). This technique is particularly effective in isolating basic drugs by increasing their water solubility and facilitating their separation from the hair matrix [105]. Drugs including cocaine, amphetamines, methamphetamines and synthetic opioids can be efficiently extracted using this method. Digestion in a basic NaOH solution can be particularly efficient for the extraction of cannabidoids and amphetamines [104].

In contrast to chemical digestion methods, solvent extraction methods do not degrade the keratinous matrix but instead dissolve and extract the analytes from it. The solvents used might be either organic or water based. Aqueous buffers such as phosphate buffer can be used to successfully extract ketamine or benzodiazepines. Organic solvent extraction on the other hand is the most frequently employed technique in drug analysis due to its efficiency in isolating almost all drugs. Methanol is the most widely used organic solvent and acts by entering the hair shaft, causing swelling and diffusion of the substrate, as well as dissolution of neutral and lipophilic compounds [104,105]. It is used to recover cocaine, benzodiazepines, amphetamines, methamphetamines, opioids, as well as hallucinogens and other synthetic cathinones.

Other approaches, such as the liquid-liquid extraction (LLE) technique, employ two immiscible liquids, an aqueous and an organic one and can be useful to isolate lipophilic drugs. Similarly, during the solid-liquid extraction, hair is embedded in a solvent and the analyte diffuses over time. Finally, mechanically assisted methods including sonication, pressurized liquid extraction and microwave assisted extraction can be used along with chemical or solvent digestion methods to enhance the extraction efficiency.

##### 1.2.1.1 Heavy metal exposure and toxicology

Hair has a long history as a biomarker for heavy metal exposure. Many heavy metals and trace elements are incorporated into hair during its growth, and hair often concentrates some of these elements at higher levels than blood [106,107]. This makes hair an attractive tissue for assessing chronic exposure to heavy metals. Hair analysis for heavy metals is routinely used in environmental health studies and forensic investigations of poisoning. Metals with density higher than 5 g/cm^2^ including lead (Pb), cadmium (Cd), mercury (Hg), zinc (Zn), copper (Cu), chromium (Cr), nickel (Ni) and antimony (Sb), are classified as heavy metals, while arsenic (As) and aluminum (Al), although less dense, also cause toxicity and are included in the same category. Heavy metals such as iron (Fe), Zn, Cu, Pb, As and Hg are naturally found in human hair, as they are incorporated during its growing phase. In addition to these commonly found elements, hair could also serve as a potential source for mineral analysis [106].

Some of the earliest forensic applications of hair involved heavy metal detection. A classic example is arsenic: hair from victims of suspected As poisoning in the 19^th^ century was analyzed to confirm chronic As ingestion [3]. Because As and other metals remain locked in hair long after exposure, hair can retrospectively reveal poisoning even when other evidence is gone. Lead was another metal historically measured in hair, especially during the era of leaded gasoline and paint. Lead accumulates in tissues, especially bones and teeth, following inhalation or ingestion, and can only be excreted or slowly metabolized. Endogenous Pb can be absorbed by the hair root through the blood [[108], [109], [110]]. However, using hair to trace lead and other heavy metals in everyday clinical practice has raised concerns, mainly due to the difficulty of removing external contamination through the washing process and the lack of reference cutoff values.

Testing hair for Pb poisoning in children has been considered to be inadequate due to low sensitivity rates and high false negative results, necessitating whole blood analysis for a definite diagnosis [110]. Nevertheless, advances in analytical methods and careful sampling could improve the performance of hair testing as a screening tool. Notably, examining hair above the scalp, which is more prone to environmental lead exposure, may not accurately reflect the actual accumulation of Pb in the body. In contrast, hair taken from below the scalp has shown a positive correlation with Pb blood concentration suggesting the potential use of this method as reliable screening tool for the general population [111].

Hair analysis has been used to assess environmental pollution in populations with high exposure. For example, in an industrial area in China known for increased heavy metal pollution, human hair and blood was screened for the presence of 12 elements. Despite the challenges in detecting certain elements, such as Cr, a strong correlation was found between the heavy metal load and the duration of exposure. These was particularly evident in the levels of Pb, Hg, Al, Cd, Zn and Sb in hair, with the highest concentrations observed in people older than 35 years [106]. Similarly, hair Cr and Pb concentrations have been found elevated in the hair of tannery workers [112].

##### 1.2.1.2 Heavy metal bio-tracing in cardiovascular diseases

Atherosclerosis is a chronic inflammatory disease that involves the vascular endothelium, immune cells, lipids and debris, resulting in plaque formation. Accumulating atherosclerotic lesions are responsible for an increased risk of coronary artery disease, as well as carotid and peripheral artery diseases, high blood pressure and chronic kidney failure, all of which lead to significant mortality rates. In an effort to identify inflammatory markers that could potentially serve as prognostic factors, trace elements have been detected in the serum of patients with inflammatory diseases [113,114].

Chronic inflammation may be traceable through hair. In a series of studies, Urbanowicz et al. identified heavy metal patterns in the hair of patients with coronary disease. Specifically, when patients with coronary artery disease were examined for heavy metal concentrations in the hair, a significant correlation between the systemic inflammatory index and the levels of Li, Fe, Cr and Sb concentrations was observed [15]. Notably, Na as well as the trace elements Cr, V and Ni were positively associated with one or two vessel occlusions, but not with three vessel disease. In addition, Mg and Ca concentrations were inversely correlated with advanced coronary disease [14].

Distinct heavy metal patterns have been identified between left and right sided atherosclerotic lesions. One study found that atherosclerosis in the main coronary artery was associated with elevated hair concentrations of Ni, Zn, and Sb. In the two main branches of the coronary artery, right coronary artery disease showed negative correlations with Mg and Sr, while both the left anterior descending and circumflex arteries exhibited similar associations with Sr. Additionally, lesions in the left anterior descending artery were linked to Cr, Na, As, and Mo, whereas occlusion in the circumflex artery was positively associated with Cr, Na, and As, and also showed links to K and Ni levels in hair [13]. Plaque location also influenced trace metal distribution in hair, with differences in Cr, Cu, and Zn observed in patients with coronary versus carotid artery disease [16]. Furthermore, low hair concentrations of Zn, Mn, and Cu, combined with elevated Pb levels, were found to differentiate deep vein thrombosis (DVT) patients from healthy controls [18].

Heavy metals have been demonstrated to be altered in other Inflammatory diseases in a few preliminary studies. For example, Cd, Ni and Pb were found to be higher in hair samples from patients diagnosed with rheumatoid arthritis, compared to controls [54]. Similarly, significant differences in Fe, Se, Mn concentrations were reported among individuals with inflammatory bowel disease, Crohn's disease, and healthy subjects [54]. In general, higher hair Mn levels were associated with better overall health status, whereas lower Zn concentrations were more frequently observed in older individuals with poorer health outcomes [115].

#### 1.2.2 Heavy metal analysis in cancer

Emerging evidence suggests that cancer may leave a detectable signature in hair strands, a phenomenon explored in several preliminary studies. Despite the typically small sample sizes, distinct heavy metal accumulation patterns have been found to correlate significantly with various cancer types. Metal bio-tracing analyses have been conducted in patients with breast, prostate, gastrointestinal, head and neck, and lung cancers, revealing notable differences in trace element profiles when compared to healthy controls (Table 1) [[22], [23], [24], [25], [26]].

Metals in hair from patients with different malignancies were investigated by Pasha and colleagues, who characterized the distribution of 18 metals in scalp hair samples in 111 patients with cancer and 113 controls [23]. Cancer patients demonstrated higher levels of Ca, Na, Zn, Mg, Fe, Sr, and K compared to controls, while another study showed decreased levels of selenium, zinc and copper [22]. They followed up on this finding by studying patients with malignant breast cancer and again found higher concentrations of Ca, Na, Mg, Zn, Sr, Fe and K in the scalp hair compared to patients with benign breast lesions and controls [116]. Skalny and colleagues investigated 103 patients that had benign breast disease, 107 patients with Stage II breast cancer and 100 women controls and demonstrated that the breast cancer patients had significantly higher hair Cr and V levels, as well as reduced Cu and Mn content as compared to both benign breast disease patients and controls [25].

In breast and lung cancer patients, Cihan and colleagues noted distinct patterns of heavy metals and trace elements in comparison to healthy controls. In both breast and lung cancers, statistically significant difference in the concentrations of 11 heavy metals was found, including Ca, Cd, Co and Zn (Table 1). To the contrary, increased As, B, Cs, Gd, Mn, Pb and Se were only positively correlated with breast cancer, while Bi, Cr, Cu, Fe, Ga, Hg, K, Rb, Rh, Ti and V were elevated in patients with IIIB non-small cell lung cancer compared to controls [24,29]. In another study enrolling 56 patients with non-small lung cancer, As and Cd were found to be elevated compared to 72 healthy controls. However, the difference did not reach statistical significance [28].

In a study involving 100 participants investigating the relationship between heavy metal concentrations and prostate cancer, elevated levels of Mn, Fe, and Cu were observed in prostate cancer patients, while Se and Zn levels were comparatively lower [26]. Similarly, in a small cohort of patients with gastrointestinal cancer, mean hair concentrations of Zn, Fe, Pb, Cu, and Cd were significantly higher than those measured in healthy controls [30].

Taking together, the available evidence suggests potential correlations between specific heavy metals and malignancy. However, the limited number and small sample sizes of existing published studies constrain the ability to draw definitive conclusions.

#### 1.2.3 Evaluating chronic stress and health using cortisol levels

Stress is reflected in cortisol levels, and hair analysis has gained attention in recent years as a method for measuring chronic cortisol exposure. Cortisol levels fluctuate significantly throughout the day and are highly influenced by acute stress. Therefore, hair's stability and its ability to record the accumulation of hormones over an extended period offer a distinct advantage in exploring disturbances in the hypothalamic-pituitary-adrenal (HPA) axis.

Hair cortisol levels have been tested in a variety of conditions, both mental and physical, that are known to be associated with chronic stress. Elevated cortisol levels have been observed in metabolic diseases such as Cushing's syndrome, diabetes mellitus, metabolic syndrome, several cardiovascular diseases and gastrointestinal disturbances, as well as bipolar and major depressive disorders [55]. Conversely, generalized anxiety and panic disorders are typically associated with decreased hair cortisol levels [6].

Hair cortisol testing presents a promising tool for both the diagnosis and treatment monitoring of hypercortisolism. One key advantage of hair as a biologic matrix for retrospective timeline analysis lies in its ability to indicate the onset of the disease through increased cortisol levels in the hair segment corresponding to specific past time points. This approach may be particularly valuable in diagnosing cyclic Cushing's syndrome, in which episodes of hypercortisolism can be unpredictable and may remain undetected in blood or urine tests. Additionally, patients undergoing treatment for adrenal insufficiency with hydrocortisone, a molecule identical to endogenous cortisol, exhibited higher average hair cortisol levels. These levels correlate positively with administered hydrocortisone doses, providing a potential means of detecting overtreatment and facilitating individualized dose optimization when considered alongside clinical presentation [6].

Cardiovascular risk has also been evaluated through hair cortisol testing. One of the major adverse effects of chronic glucocorticoid use or hypercortisolemia is an increased risk for cardiovascular events, along with metabolic disfunctions, such as obesity, dyslipidemia, metabolic syndrome and hypertension [117]. However, monitoring blood or urine cortisol levels has failed to establish a correlation between cortisol levels and cardiovascular risk profiles. Hair cortisol levels have been found to be significantly elevated in individuals with type 2 diabetes mellitus and an increased risk of cardiovascular disease [6].

Many studies are investigating cortisol exposure as a means of evaluating mental health and stressful life events. Exposure to significant life events appears to be reflected in hair cortisol levels in young adults [47]. One study, for example, examined hair cortisol levels as a marker of maternal prenatal stress, which is linked to adverse pregnancy and birth outcomes, finding an association between hair cortisol levels and perceived stress among pregnant women. In addition, hair cortisol levels were found to be lower in women with preterm labor, although there is no conclusive evidence linking preterm labor rates to perceived stress in these women [61].

##### 1.2.3.1 Emerging multi-omics biomarkers in hair

The field of hair diagnostics is rapidly evolving with the integration of multi-omics technologies, enabling comprehensive molecular analyses within a single hair sample. In addition to targeted assessments of specific compounds such as drugs, heavy metals, or cortisol, untargeted metabolomic and proteomic approaches are increasingly being applied to hair to uncover novel disease biomarkers. These high-throughput strategies have the potential to facilitate the identification of complex molecular signatures—spanning metabolites, proteins, and gene-related products—that may signal disease presence or predisposition, prior to the onset of clinical symptoms.

*Metabolomics in neurodegenerative disease:* Several studies linked metabolites from hair to Alzheimer's disease [[118], [119], [120], [121]]. Chang and colleagues utilized untargeted metabolomic profiling of hair from an Alzheimer's disease mouse model and from human patients using high-resolution mass spectrometry [122]. The study revealed distinct metabolic alterations in the hair of Alzheimer's disease mice before the hallmark amyloid plaques appeared in the brain. Specifically, changes in pathways such as arachidonic acid metabolism and sphingolipid metabolism were observed in the hair metabolites of very young transgenic mice, suggesting early pathophysiological changes [122]. Correlating these findings with human samples, a combination of two hair metabolites (l-valine and arachidonic acid) could distinguish Alzheimer's disease patients from healthy controls with approximately 80 % sensitivity and specificity [122]. This proof-of-concept indicates that hair may carry a metabolic fingerprint of neurodegeneration. If validated in larger cohorts, a simple hair test could someday aid in screening for Alzheimer's or other neurodegenerative diseases years before overt cognitive decline, enabling earlier intervention.

*Proteomics in psychiatry*: Although hair predominantly consists of keratins, advancements in mass spectrometry techniques now enable the identification of hundreds of proteins from hair extracts or even directly from the hair shaft [[123], [124], [125], [126]]. A recent preprint has expanded this application to the field of mental health, exploring hair proteomics as a diagnostic tool for mental health conditions [127]. Proteomic profiling of hair has been explored in patients with non-suicidal self-injury disorder, a condition frequently associated with severe emotional dysregulation). In a recent study, over 600 hair proteins were quantified and analyzed using machine learning, resulting in a classification accuracy of approximately 84 % when distinguishing patients from healthy controls. Notably, the proteomic model outperformed traditional markers, including hair cortisol and panels of known stress-related proteins. Key discriminative proteins were linked to biological pathways involved in pain perception, oxidative stress, and ribosomal function, the latter potentially reflecting mechanisms implicated in depression [[128], [129], [130]]. These findings suggest that subtle, temporally integrated alterations in the hair proteome, may reflect chronic physiological stress or underlying molecular alterations associated with psychiatric conditions. With further validation and refinement, a “hair proteomic biopsy” could serve as a non-invasive tool for the diagnosis or monitoring of disorders such as chronic anxiety and depression, offering an extended temporal resolution compared to conventional blood-based biomarkers.

*Cancer Biomarkers in Hair*: Beyond heavy metal analysis, broader metabolomic/proteomic fingerprints for cancer are also being explored [131,132]. Tumors are known to release a range of metabolites and proteins, which may become incorporated into hair either through bloodstream delivery to the follicle or via diffusion from sweat and sebaceous gland secretions onto the hair shaft [125,[133], [134], [135], [136]]. In particular, aggressive cancers may induce distinct biochemical alterations in hair composition. For example, cachexia or tumor-secreted factors can disrupt systemic amino acid metabolism levels potentially leading to detectable shifts in the levels of specific amino acids or their derivatives in hair [137,138].

*Hair genomics and epigenetics*: Another emerging area is the use of hair follicles as a source of DNA/RNA to detect genomic or epigenomic biomarkers of disease [[139], [140], [141]]. For example, cancer detection and monitoring via “liquid biopsy” of circulating tumor DNA is an area under of intense investigation. Analogously, DNA shed into hair [142] or present in hair roots [143] may record tumor mutations for certain cancers. Epigenetic modifications, e.g., DNA methylation patterns, have been studied in blood for cancer and aging markers; these can also be measured in hair follicle DNA [144,145]. Although currently primarily a research tool, hair-based analysis holds the potential to evolve into a comprehensive diagnostic platform—offering simultaneous biochemical, genetic, and epigenetic readouts from a single sample to enable holistic assessment of an individual's health status.

#### 1.2.4 Analytical techniques for hair analysis

Unlocking the diagnostic potential of hair has been made possible by advances in analytical chemistry that allow detection of minute quantities of substances embedded in the hair matrix. Early hair analysis relied on relatively crude assays such as colorimetric tests or radioimmunoassays, but modern techniques—especially those based on Mass Spectrometry (MS), and related methods—have greatly increased sensitivity, specificity and the range of detectable analytes. Here we outline key analytical techniques used for hair, including recent innovations (Table 2).

**Table 2:** Comparison of analytical techniques for hair analysis.

Technique	Principle	Use
***Gas Chromatography-Mass Spectrometry (GC-MS)***	Separation of volatile compounds, followed by mass spectrometry	Detection of small, volatile, thermally stable molecules
*Liquid Chromatography– Mass Spectrometry (LC-MS)*	Separation of compounds in liquid phase, followed by mass spectrometry	Detection of polar, non-volatile, thermally unstable molecules
*High- Resolution Mass Spectrometry (HRMS)*	Determination of the atomic masses of organic and inorganic molecules	Detection of unknown compounds with high accuracy
*Inductively Coupled Plasma Mass Spectrometry (ICP-MS)*	Ionization of elements in a plasma, followed by mass spectrometry	Quantification of metals and toxins, measurement of isotope ratios
*Inductively Coupled Plasma Optical Emission Spectroscopy (ICP-OES)*	Measurement of light absorption from atoms excited in plasma	Cost-effective for multi-element detection for elements at higher concentrations
*Atomic Absorption Spectroscopy (AAS)*	Measurement of light absorption from atoms in a flame	Measurement of specific metals, one at a time, at low cost
*Time-of-Flight Secondary Ion Mass Spectrometry (ToF-SIMS)*	Measurement of secondary ions ejected from the sample surface	Surface analysis of trace elements, drugs, or metabolites in hair
*Matrix-Assisted Laser Desorption/Ionization Mass Spectrometry (MALDI-MS)*	Laser-induced desorption and ionization, followed by mass spectrometry	Analysis of large biomolecules
X-ray fluorescence (XRF)	Measurement of X-ray fluorescence emitted by elements	Detection of heavy metals without complex preparation
*Scanning Electron Microscopy (SEM)*	Imaging of sample surface with electron beams	High-resolution imaging of the hair surface
*Transmission Electron Microscopy (TEM)*	Imaging after beam of electrons passes through the sample	Extremely high resolution; can analyze ultrastructure

*Chromatography-mass spectrometry:* The gold standard for detecting organic compounds in hair involves chromatographic separation coupled with mass spectrometric detection. Two common approaches are *Gas Chromatography–Mass Spectrometry (GC-MS)* and *Liquid Chromatography–Mass Spectrometry (LC-MS)*. Depending on the targeted biomarker, different types of methods may be employed. GC-MS is suitable for small, volatile and thermally stable molecules, while LC-MS may be more useful for testing polar and thermally unstable samples. In addition, LC-MS is superior in analyzing a broader range of metabolites and offers an easier and faster sample preparation compared to GC-MS [146].

Despite the differences, both methods were validated with excellent linearity and low detection limits in drug analysis, and have been, for example, applied for fentanyl detection in hair [147]. Similarly, a sensitive GC-MS method has been developed for detecting major drugs of abuse, including cocaine, morphine, and amphetamines, while LC-MS has been used to analyze long-term steroid metabolism and quantify multiple steroid classes, including glucocorticoids, androgens, progestogens, and estrogens in human scalp hair [148]. Modern LC-MS systems often using high-performance liquid chromatography (HPLC) separations and tandem mass spectrometers can detect a broad range of compounds with minimal sample preparation.

An emerging trend is the use of High-Resolution Mass Spectrometry (HRMS), such as time-of-flight or Orbitrap MS detectors, in untargeted metabolomic or proteomic studies of hair. HRMS provides exact mass measurements that facilitate the identification of unknown biomarkers and can detect hundreds of compounds in a single run. This has recently been applied to discover novel disease biomarkers in hair, such as metabolic signatures associated with Alzheimer's disease [122]. The high mass accuracy and resolution of HRMS are critical for confidently distinguishing compounds within the complex chemical mixture extracted from hair.

*Inductively Coupled Plasma Mass Spectrometry:* Many diagnostic applications of hair involve measuring inorganic elements such as toxic heavy metals or essential trace minerals. The primary tool for multi-element analysis is *Inductively Coupled Plasma Mass Spectrometry (ICP-MS)*, which can detect trace levels of metals and metalloids with high sensitivity. ICP-MS can quantify toxic elements like lead, mercury, arsenic, as well as nutritionally relevant metals like zinc and iron [149,150]. It also allows measurement of isotope ratios—for example, isotopic signatures of strontium in hair have been used to infer geographic region or diet [81]. However, ICP-MS alone cannot distinguish between ionic species and therefore separate toxic from non-toxic valences of the same element. For example, the inorganic arsenic species arsenite (As^3+^) and arsenate (As^5+^) are highly toxic and carcinogenic, while organic species such as arsenobetaine, arsenocholine, monomethylarsonic acid and dimethylarsinic acid are generally considered non-toxic. To accurately assess the health risks associated with elemental exposure, it is essential to separate these species prior to detection. This can be achieved by coupling ICP-MS with a separation technique such as ion chromatography (IC) or high-performance liquid chromatography (HPLC), a configuration often referred to as LC-ICP-MS. These hyphenated techniques enable the differentiation of element species or valences before they are atomized and ionized for mass spectrometric detection [151].

A variant technique, Laser Ablation ICP-MS (LA-ICP-MS), has advanced the capability of obtaining spatially resolved elemental profiles along single hair strands. In LA-ICP-MS, a focused laser beam ablates microscopic segments of the hair sequentially from root to tip, feeding the aerosol into the ICP-MS. This provides a continuous timeline of metal exposure with very high resolution [152]. LA-ICP-MS thus enables to time-stamp metal fluctuations, detect acute exposure events, and even distinguish external surface contamination by comparing element levels near the hair surface vs the interior core [153,154]. Such precision is particularly useful for forensic reconstructions of poisoning or for monitoring short-term changes in exposure that segmented bulk analysis might average out [154,155].

Other techniques for elemental analysis include *Inductively Coupled Plasma Optical Emission Spectroscopy (ICP-OES)* and *Atomic Absorption Spectroscopy (AAS)*. ICP-OES is cost-effective for multi-element detection but less sensitive than ICP-MS, making it suitable when elements are at higher concentrations [22,156]. Flame AAS and electrothermal AAS can measure specific metals, one at a time, and are used in some toxicology labs due to lower cost, albeit with lower sensitivity compared to ICP-MS.

*Other spectroscopic and imaging techniques:* Beyond mass spectrometry, several spectroscopic methods may help analyze the composition or structure of hair. *Time-of-Flight Secondary Ion Mass Spectrometry (ToF-SIMS)* is a surface analysis technique that bombards the hair surface with ion beams and analyzes ejected secondary ions, mapping molecular and elemental distribution on the hair. It has been used alongside MALDI to examine whether drug residues on hair are due to external contamination or internal incorporation [157].

*Matrix-Assisted Laser Desorption/Ionization Mass Spectrometry (MALDI-MS)* can directly analyze large biomolecules (like peptides, proteins, or pigments) from hair surfaces or cross-sections. A combined ToF-SIMS and MALDI study demonstrated that even if a hair is externally contaminated by drugs, the contaminants are mostly confined to the outer layers, whereas the internally incorporated drug can still be distinguished in the hair cortex [157]. For non-destructive elemental screening, X-ray fluorescence (XRF) can rapidly detect certain heavy metals in hair without complex preparation, although its sensitivity is generally lower than ICP-based methods [158].

*Scanning Electron Microscopy (SEM)* provides high-resolution imaging of the hair surface and can reveal damage, deposits, or abnormalities. SEM has been used to detect subtle hair shaft damage from cosmetic treatments (bleaching, dye, heat) that are not visible by light microscopy [159]. Such damage might affect how substances penetrate or bind to hair. *Transmission Electron Microscopy (TEM)* can visualize internal hair structures at the nanoscale, which has been used in research on hair disorders, for example, showing disrupted cortex structure and melanin granule changes in alopecia areata [160]. While SEM/TEM may not be suitable for routine chemical analysis, they can corroborate physical evidence of hair changes due to disease or external factors.

*Immunoassays and other emerging technologies:* Immunoassays can be applied to hair extracts for specific proteins or hormones. For example, enzyme immunoassays for cortisol have been used to measure hair cortisol level as a stress biomarker [47]. Immunoassays are less common for hair than for fluids, but they offer a simple and relatively affordable option for certain analytes if validated [55]. As noted above, emerging techniques also include hair transcriptomics and genomics. While the hair shaft (keratinized) has no DNA, the attached follicle cells can provide material for hair follicle genomic analysis. This has seen use in forensic genetics (DNA profiling from hair roots) [82] and is being explored in research for epigenetic markers of stress or aging detectable in hair DNA. For example, DNA methylation patterns in hair follicles might serve as an epigenetic “clock” of biological aging or be indicative of disease-related changes in gene regulation [161,162]. Additionally, detecting pathogens or their DNA/RNA in hair is a nascent area [163]. Though not yet in mainstream practice, these genomic and transcriptomic analyses of hair could broaden the diagnostic scope of hair beyond the biochemical composition of the shaft.

## 2 Discussion

The value of hair as a diagnostic medium in drug and toxin exposure is well established. Recent research has established associations between heavy metal accumulation in hair and various diseases. Beyond the observed correlations between elemental patterns and the two leading causes of mortality—cardiovascular disease and malignancies—other medical conditions have been linked to specific trace element imbalances. The measurement of other analytes, proteins, and nucleic acids to detect and monitor disease shows promise. Although a larger number of patients and studies are necessary to draw robust conclusions, hair analysis potentially offers an easily accessible tool for widespread population screening for many different diseases. However, despite its potential, hair analysis presents several challenges and limitations.

One of the major challenges in hair analysis is the risk of false positive results caused by passive exposure or environmental contamination. Elements that are found in lower concentrations in hair are more prone to external contamination, which complicates the correct interpretation of the results. Usual contamination sources include atmospheric pollution, environmental heavy metal toxicity, diet and/or water, which may be distinguished from the actual signal using different isotope systems. For example, δ^34^S value has been used to monitor individual's geographical origin due to the distinct patterns of S isotopes found in their hair based on their location and the diet they consume [71,80,164,165]. In addition, oxygen isotopes can reflect the source of drinking water [166]. When analyzing hair aiming for drug detection, a washing step prior to analysis may help mitigate contamination, but may also remove the desired drug from both the surface the interior of the hair strand [5,166].

Another challenge is setting a detection threshold for a target substance. It is often not possible to precisely determine the dose or duration of drug administration, due to a lack of comparative data and the significant variability in drug concentration within hair from different anatomical sites or among individuals with differing morphological and social characteristics. For example, chemical hair treatment or hair coloring may be a confounding factor in the measured drug or metabolite concentration [167]. Studies have shown that chemical treatments can lead to lower levels of certain drugs (e.g., morphine, cocaine, codeine, diazepam), with cortisol also being subject to this trend. Additionally, melanin content in hair can influence drug concentration measurements, as darker hair tends to accumulate larger amounts of alkaline substances [5,168].

Hair has the potential to serve as a valuable biomarker for monitoring physiological and pathological changes in the body due to its non-invasive and non-sterile collection process, as well as its stability without requiring preservatives prior to analysis. Additionally, most hair-based assays do not necessitate the extraction of hair follicles, which means that whole-genome DNA is not collected (a concern for many people). Consequently, hair remains an underutilized biological material for medical and diagnostic applications.

## CRediT authorship contribution statement

**Venetia A. Florou:** Writing – review & editing, Writing – original draft, Visualization, Resources, Investigation. **Auraya Manaprasertsak:** Writing – review & editing, Investigation. **Maria Slyusarenko:** Writing – review & editing. **Sarah R. Amend:** Writing – review & editing. **Julhash U. Kazi:** Writing – review & editing, Investigation. **Emma U. Hammarlund:** Writing – review & editing, Supervision. **Kenneth J. Pienta:** Writing – review & editing, Writing – original draft, Supervision, Conceptualization.

## Funding

No funding was received for this article.

## Declaration of competing interest

The authors declare no competing interests.
